# Impaired interhemispheric synchrony in patients with iridocyclitis and classification using machine learning: an fMRI study

**DOI:** 10.3389/fimmu.2024.1474988

**Published:** 2024-12-16

**Authors:** Yan Tong, Zhi Wen, Xin Huang

**Affiliations:** ^1^ Department of Ophthalmology and Visual Sciences, The Chinese University of Hong Kong, Hong Kong, Hong Kong SAR, China; ^2^ Department of Radiology, Renmin Hospital of Wuhan University, Wuhan, Hubei, China; ^3^ Department of ophthalmology, Jiangxi Provincial People’s Hospital, The First Affiliated Hospital of Nanchang Medical College, Nanchang, China

**Keywords:** interhemispheric integration, iridocyclitis, support vector machine, machine learning, fMRI

## Abstract

**Background:**

This study examined the interhemispheric integration function pattern in patients with iridocyclitis utilizing the voxel-mirrored homotopic connectivity (VMHC) technique. Additionally, we investigated the ability of VMHC results to distinguish patients with iridocyclitis from healthy controls (HCs), which may contribute to the development of objective biomarkers for early diagnosis and intervention in clinical set.

**Methods:**

Twenty-six patients with iridocyclitis and twenty-six matched HCs, in terms of sex, age, and education level, underwent resting-state functional magnetic resonance imaging (fMRI) examinations. The study employed the voxel-mirrored homotopic connectivity (VMHC) technique to evaluate interhemispheric integration functional connectivity indices at a voxel-wise level. The diagnostic efficacy of VMHC was evaluated using a support vector machine (SVM) classifier, with classifier performance assessed through permutation test analysis. Furthermore, correlation analyses was conducted to investigate the associations between mean VMHC values in various brain regions and clinical features.

**Results:**

Patients with iridocyclitis exhibited significantly reduced VMHC signal values in the bilateral inferior temporal gyrus, calcarine, middle temporal gyrus, and precuneus compared to HCs (voxel-level P < 0.01, Gaussian Random Field correction; cluster-level P < 0.05). Furthermore, the extracted resting-state zVMHC features effectively classified patients with iridocyclitis and HCs, achieving an area under the receiver operating characteristic curve (AUC) of 0.74 and an overall accuracy of 0.673 (P < 0.001, non-parametric permutation test).

**Conclusion:**

Our findings reveal disrupted interhemispheric functional organization in patients with iridocyclitis, offering insight into the pathophysiological mechanisms associated with vision loss and cognitive dysfunction in this patient population. This study also highlights the potential of machine learning in ophthalmology and the importance of establishing objective biomarkers to address diagnostic heterogeneity.

## Introduction

Uveitis, a prevalent inflammatory ocular disease that poses a significant risk to vision, is reported to have an annual incidence ranging from 50% to 92% in western countries (1). Iridocyclitis accounts for around 50% of uveitis cases; it is characterized by inflammation limited to the anterior segment of the eye, affecting the iris and ciliary body. Typical symptoms include eye pain, redness, miosis, blurred vision, and photophobia (2). If left untreated, iridocyclitis can lead to a variety of vision-threatening ocular complications, such as cystoid macular edema, secondary glaucoma, anterior synechiae, and high intraocular pressure, all of which may result in transient or permanent vision loss (3). Additionally, iridocyclitis is commonly associated with systemic immune-mediated diseases linked to human leukocyte antigen-B27, including inflammatory bowel disease, ankylosing spondylitis, and reactive arthritis (4). While systemic corticosteroid therapy can effectively reduce ocular inflammation during flare-ups (5), it often proves inadequate for many patients with chronic iridocyclitis and fails to prevent future relapses. Currently, the pathophysiology of iridocyclitis remains unclear.

Research into the pathogenesis of iridocyclitis has incorporated imaging studies to identify relevant structural and functional changes. Optical coherence tomography assessments have demonstrated that iridocyclitis can affect retinal thickness, central foveal thickness, and retinal nerve fiber layer thickness (6, 7). The progress in neuroimaging methods has introduced functional brain imaging as a valuable tool for investigating the neural mechanisms underlying iridocyclitis and its associated symptoms. Our previous study found notable reductions in the amplitudes of low-frequency fluctuation in the right calcarine region among iridocyclitis patients compared to healthy controls (HCs) (8). Additionally, patients with iridocyclitis exhibited diminished regional homogeneity values in the inferior occipital gyrus, superior occipital gyrus, and bilateral calcarine regions (9). Furthermore, there was markedly reduced functional connectivity between the primary visual cortex (V1) and the superior occipital gyrus, as well as between V1 and the bilateral calcarine (10). However, the specific aspects of homotopic connectivity, where corresponding areas in the two hemispheres of the brain communicate, remain largely unexplored. Thus, we hypothesize that patients with iridocyclitis exhibit abnormal interhemispheric synchrony within specific brain regions, including the alterations in visual regions.

Interhemispheric synchronization in the human brain is primarily facilitated by the corpus callosum, which connects the left and right hemispheres, allowing for coordinated neural activity. Functionally, it plays a critical role in cognitive processing, sensory integration, emotional regulation, motor coordination, and memory. Interhemispheric synchronization of the visual cortex has been documented across various species (11, 12). The robust homotopic connectivity of the visual stream is a vital for integrating visual information in the human brain (13). Resting-state functional magnetic resonance imaging (rs-fMRI), utilizing blood oxygenation level-dependent signals, has been extensively employed to unveil patterns of interhemispheric connectivity in the brain *in vivo*. Zuo et al. introduced the voxel-mirrored homotopic connectivity (VMHC) technique, which serves as a dependable and replicable voxel-wise metric for analyzing rs-fMRI data (14). VMHC quantifies resting-state functional connectivity between each voxel in one hemisphere and its mirrored counterpart in the other (14). This approach has been effectively utilized to investigate disruptions in homotopic connectivity in various neurological and ophthalmological conditions, including blepharospasm (15)., glaucoma (16), late blindness (17), schizophrenia (18), and insomnia (19). The advantages of VMHC include its sensitivity to subtle changes in functional connectivity, its capacity to detect interhemispheric communication disruptions, and its robustness and reproducibility across studies. Given these strengths, the VMHC technique may provide valuable insights into the interhemispheric disruptions associated with iridocyclitis, paving the way for future research into the underlying mechanisms in this disease.

In recent years, machine learning, a subset of artificial intelligence, has been increasingly applied in clinical settings to enhance predictive diagnostics and improve treatment strategies. Within the field of ophthalmology, machine learning has demonstrated significant promise in integrating multimodal neuroimaging data and analyzing both structural and functional brain changes at the individual level (20, 21). These methods offer advantages such as improved pattern recognition, automated feature extraction, and the integration of multimodal data, facilitating the identification of subtle alterations in brain activities that traditional methods might overlook. Furthermore, machine learning allows for the development of predictive models that support personalized treatment plans and improve diagnostic accuracy in the clinical practice.

This study aims to examine the presence of whole-brain interhemispheric connectivity changes in patients with iridocyclitis. We further employed a machine learning approach to assess whether atypical VMHC values could effectively differentiate patients with iridocyclitis from controls. The outcomes could offer valuable insights into abnormal hemispheric interactions in individuals with iridocyclitis and aid in the clinical identification of the disease.

## Materials and methods

### Participants

A total of 26 patients diagnosed with iridocyclitis and 26 healthy controls (matched for gender and age) were recruited from the Eye Center at Jiangxi Provincial People’s Hospital. Inclusion criteria for iridocyclitis patients included: (i) diagnosis of iridocyclitis in accordance with the criteria of the Standardization of Uveitis Nomenclature Working Group classification, confirmed by two specialists; (ii) absence of clinical evidence or history of other ocular conditions such as glaucoma, high myopia, age-related macular degeneration, and epiretinal membrane; (iii) no prior treatment for iridocyclitis; (iv) right-hand dominance; (v) no history of psychiatric ailments or use of psychotropic medications; (vi) no contraindications for MRI scans. The exclusion criteria for iridocyclitis patients comprised: (i) history of ocular trauma or undergone vitreoretinal and refractive surgeries; (ii) presence of systemic diseases like hypertension, diabetes, and cerebrovascular conditions or other comorbidities.; (iii) other ocular diseases presenting similar features, such as uveitis of different etiologies. All patients in our study had their eye pain effectively alleviated prior to scanning, ensuring that scans were conducted during periods of relative stability.

The inclusion criteria for healthy controls (HCs) were as follows: (i) normal visual acuity [>1.0] in both eyes; (ii) absence of ophthalmic diseases (such as optic neuritis, cataract, keratitis); (iii) no history of psychiatric disorders; (iv) right-handed dominance; (v) no contraindications to MRI scanning.

This study was performed in accordance with the tenets of the Declaration of Helsinki; it was formally approved by the institutional review board of Eye Center, Jiangxi Provincial People’s Hospital. Each participant provided written informed consent before inclusion in the study.

### Ophthalmic examination and MRI acquisition

All participants underwent a complete ophthalmic assessment. The ocular examination included slit-lamp biomicroscope, visual acuity, gonioscopy, applanation tonometry, optical coherence tomography, dilated fundus examination, and fluorescein angiography.

fMRI data were acquired by a 3.00-Tesla magnetic resonance scanner (Discovery MR 750W system; GE Healthcare) with an eight-channel head coil. Headphones and foam paddings were used to reduce scanner noise and limit the head motion. We used three-dimensional spoiled gradient-recalled echo sequences to obtain the anatomical T1-weighted images (structural MRI data); we used gradient-recalled echo-planar imaging sequences to collect whole-brain fMRI data (detailed parameters are listed in **Table 1**). During the scanning procedure, all participants were instructed to remain still, stay awake, not to think of anything in particular, and keep their eyes closed until the scan was completed. To further mitigate the impact of subjective thoughts, participants were also given proactive runs to help them acclimate to the scanning environment. The whole scanning process is around fourteen minutes.

**Table 1:** Detailed parameters setting of the MRI scanning.

Data acquisition	Brain volume sequence	Echo-planar imaging sequence
Repetition time	1,900 ms	2,000 ms
Echo time	2.26 ms	30 ms
Field of view	0 mm	1.2 mm
Gap	240 x 240 mm2	240 x 240 mm2
Acquisition matrix	256 x 256	64 x 64
Flip angle	12°	90°

Repetition time, the time interval between successive pulse sequences applied to the same slice; Echo time, the time between the delivery of the radiofrequency pulse and the receipt of the echo signal; Field of view: the size of the imaging area covered by the MRI scan; Acquisition matrix: the number of frequency and phase encoding steps used in the scan; Flip angle, the angle at which the radiofrequency pulse is applied to the spins in the magnetic field.

### fMRI data preprocessing

First, functional data were quality checked by the MRIcro software (http://www.MRIcro.com). Next, data analysis was conducted using the statistical parametric mapping version 8 (SPM8, https://www.fifil.ion.ucl.ac.uk/spm) and the Data Processing & Analysis of Brain Imaging (DPABI) toolbox, both implemented in MATLAB R2013b (The MathWorks, Inc., MA, USA; http://www.mathworks.com/products/matlab/) (22). The preprocessing steps included: (1) converting DICOM files to NIFTI format; (2) removing the first 10 functional volumes for stabilization; (3) slice timing correction for remaining fMRI images; (4) realignment for head motion correction (Functional data from participants exhibiting angular motion greater than 1.5 degrees or displacement exceeding 1.5 mm in the x, y, or z-axis were excluded from the analysis); (5) reorienting structural and functional images; (6) segmenting structural images with the DARTEL (Diffeomorphic Anatomical Registration Through Exponentiated Lie Algebra) method to create a group template; (7) normalizing to the Montreal Neurological Institute template (3 mm × 3 mm × 3 mm resolution); (8) applying 6-mm Gaussian smoothing to mitigate confounding effects; (9) regressing nuisance covariates, and (10) performing temporal band-pass filtering (0.01–0.08 Hz).

### Voxel-mirrored homotopic connectivity analysis

The functional images of each patient were subsequently aligned with the study-specific, symmetrical MNI template for the standardized measurement of VMHC. VMHC values were calculated using the REST software, with the specifics of VMHC computation detailed in a previous study (14). Individual VMHC maps were created for each participant by calculating the Pearson correlation coefficient between a specific voxel and its counterpart in the opposite hemisphere. Subsequently, the correlation values underwent Fisher z-transformation to enhance the normality of the distribution (23). The resultant zVMHC values were used for subsequent voxel-wise group comparison.

### Statistical analysis

Clinical and demographic data were analyzed using two-sample t-tests and the Chi-square test in SPSS software, version 22 (SPSS, Chicago, IL, USA). The significance threshold was set at P<0.05. Intra-group patterns of zVMHC maps were assessed through one-sample t-tests using the REST toolbox, while group differences in zVMHC were evaluated through two-sample t-tests.(voxel wise P<0.001, GRF theory connected, cluster level, P < 0.005). BrainNet Viewer (https://www.nitrc.org/projects/bnv/) was utilized to exhibit the specific brain regions corresponding to all statistically significant outcomes. Furthermore, Pearson’s correlation coefficients were computed to investigate potential associations between the mean zVMHC values across various brain regions and the clinical characteristics of patients with iridocyclitis using SPSS software.

### Multivariate pattern analysis: SVM application in neuroimaging

In our machine learning analysis, we focused on the zVMHC data to distinguish patients with iridocyclitis using the Support Vector Machine (SVM) algorithm implemented in the Pattern Recognition for Neuroimaging Toolbox (PRoNTo) within Matlab 2014b. In this study, individual participants’ zVMHC maps were utilized as inputs for the machine learning algorithm, and a feature set was prepared using whole-brain zVMHC data. The SVM algorithm in PRoNTo is based on a linear-kernel SVM for binary classification (24).

The feature selection process was carried out automatically in the “Prepare feature set” module of PRoNTo. For estimating and optimizing the algorithm, a leave-one-out cross-validation (LOOCV) approach was employed to perform SVM classifier validation. In this approach, one sample was reserved for validation while the remaining K-1 samples were utilized for training, where K represents the total sample size in the study. The LOOCV process was iterated K times, and average metrics were utilized to assess the trained model. These steps were automatically executed in the “Specify model” module of PRoNTo.

The overall accuracy, sensitivity, specificity and area under the receiver operating characteristic curve (AUC) values were calculated to evaluate the classification performance of the SVM algorithm. Furthermore, a non-parametric permutation test was used to assess the statistical significance of the classification accuracy (25). Specifically, we performed 2,000 permutations to ensure a thorough assessment of significance levels. For each permutation, we recalculated the test statistic, allowing us to compare the observed statistic against this distribution.

## Results

### Comparison of demographic and clinical characteristics

**Figure 1:**
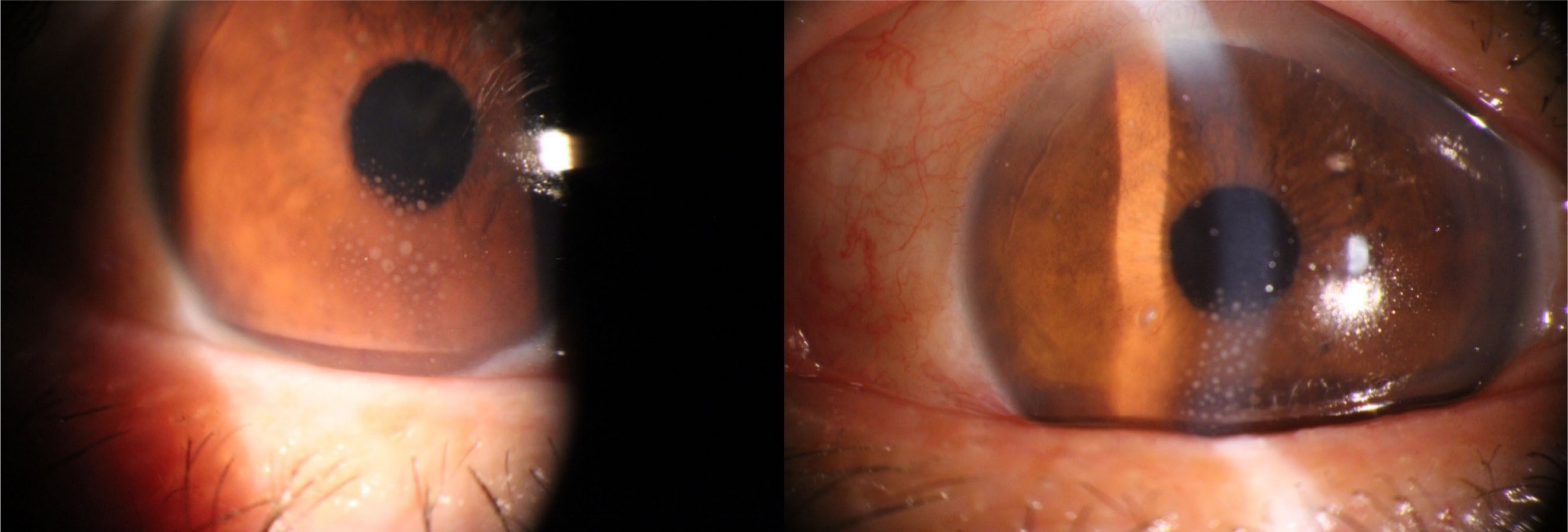
Typical ocular features in patients with iridocyclitis.

The clinical and demographic characteristics are shown in **Table 2**. There were no significant differences in sex (P=0.780), education (P=0.871), or age (P=0.97) between the patients with iridocyclitis and HCs. In contrast, significant differences were observed in bilateral best-corrected visual acuity (P<0.001). Representative anterior segment photographs of patients with iridocyclitis are shown in **Figure 1**.

**Table 2:** Clinical characteristics for HCs and patients with iridocyclitis.

	Iridocyclitis group	HC group	T-values	P-values
Sex (male/female)	14/12	15/11	N/A	0.780
Age (years) Education (years)	45.15 ± 14.95 11.04 ± 3.94	45.30 ± 13.87 11.52 ± 3.48	-0.038 -0.472	0.970 0.639
BCVA-OD	0.44 ± 0.27	1.16 ± 0.16	-11.474	<0.001*
BCVA-OS	0.43 ± 0.37	1.19 ± 0.16	-9.352	<0.001*
Duration of iridocyclitis (days)	4.43 ± 2.89	N/A	N/A	N/A
Handedness	26 R	26 R	N/A	N/A

Chi-square test for sex. Independent t-test was used for other normally distributed continuous data. Data are presented as mean ± standard deviation.

HC, healthy control; BCVA, best-corrected visual acuity; OD, oculus dexter; OS, oculus sinister; N/A, not applicable; R, right. Asterisks indicate significant differences (p < 0.05).

### Comparison of VMHC between patients with iridocyclitis and HCs

**Figure 2:**
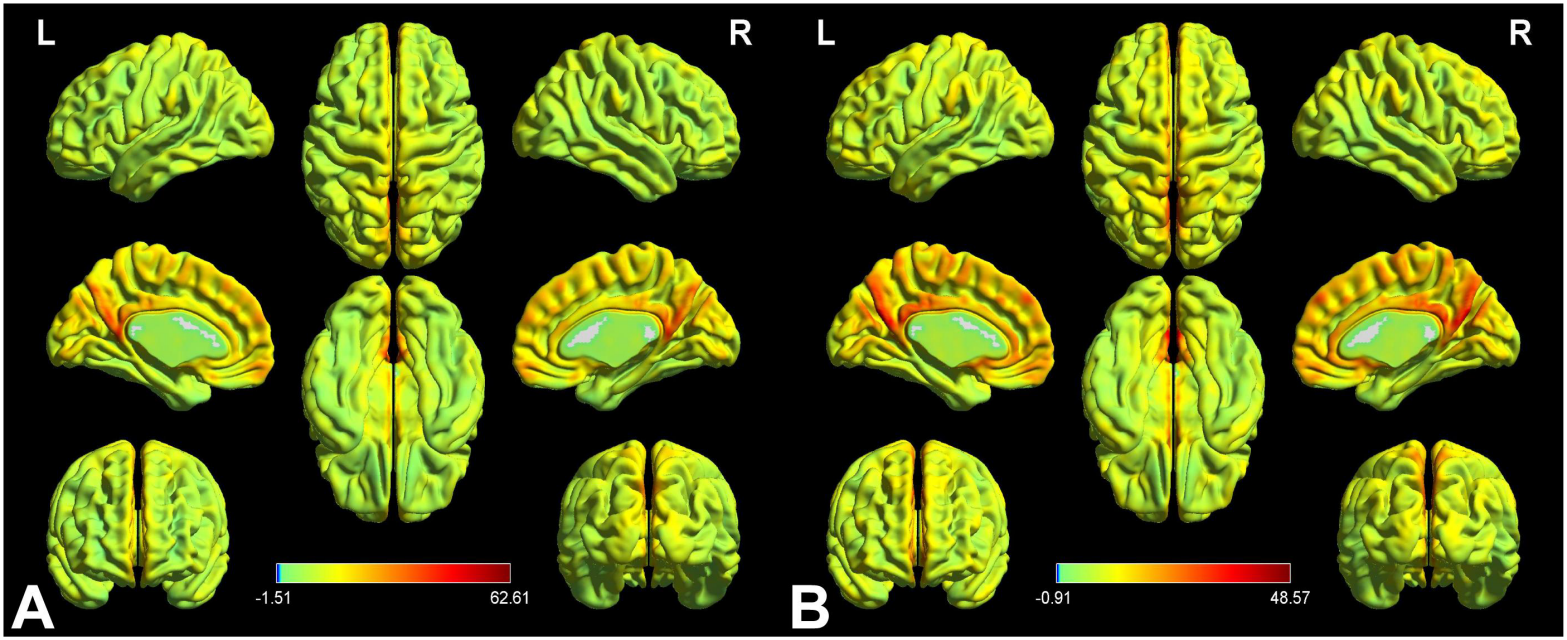
VMHC spatial maps at the group average level of HCs **(A)** and patients of iridocyclitis group **(B)** (voxel- level P<0.01, Gaussian random field correction, cluster-level P<0.05). The warm color tone suggests higher homotopic connectivity coefficients. The color bar represents the t-values. VMHC, voxel-mirrored homotopic connectivity; HC, healthy control; L, left; R, right.

**Figure 3:**
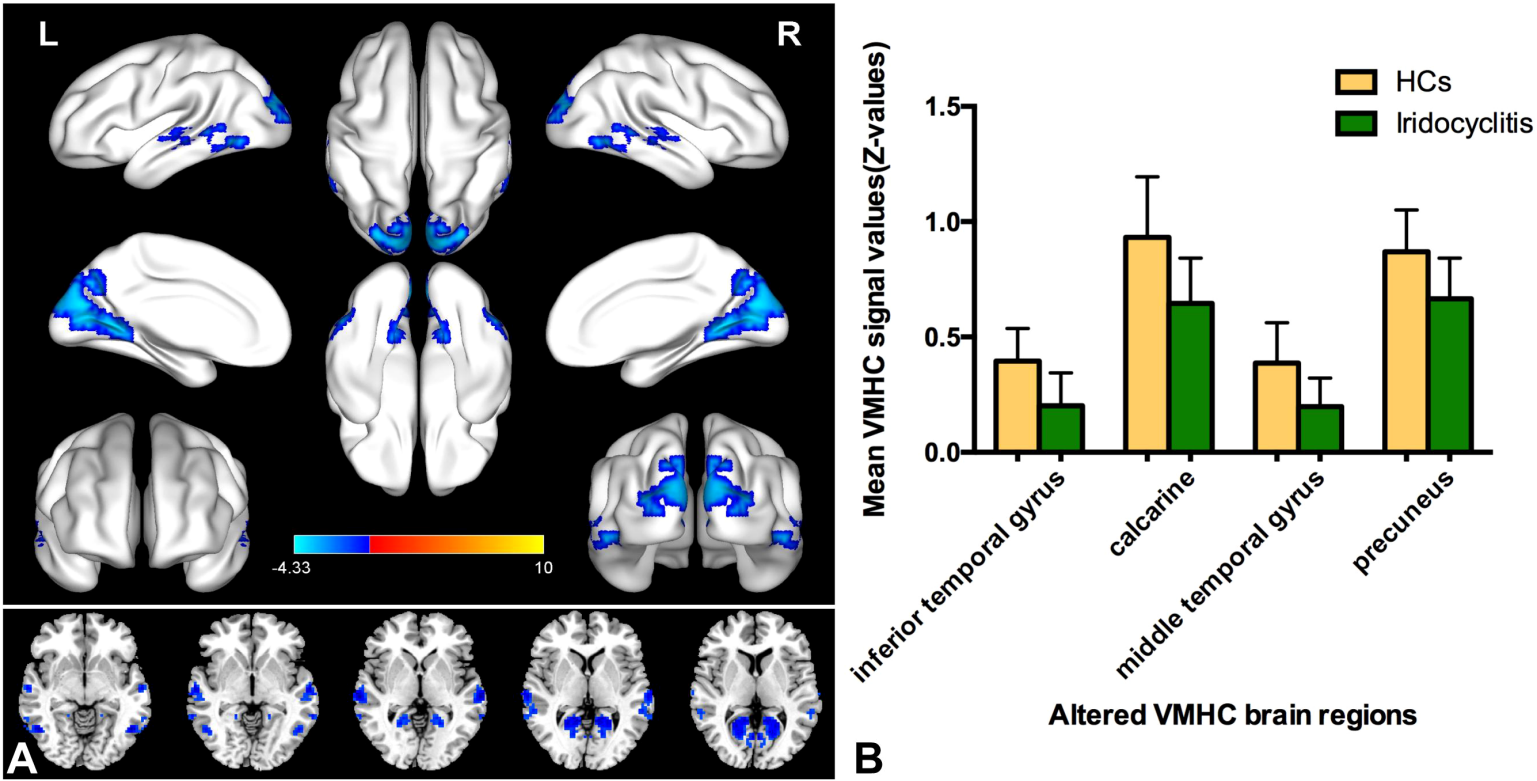
**(A)** Altered VMHC values differences in the patient group compared with the control group. Blue-indigo color indicates reduced VMHC in iridocyclitis. Patients with iridocyclitis displayed significantly reduced VMHC signal values in the bilateral inferior temporal gyrus, calcarine, middle temporal gyrus, and precuneus compared with HCs. (voxel- level P<0.01, Gaussian random field correction, cluster-level P<0.05). VMHC, voxel-mirrored homotopic connectivity; HC, healthy control; L, left; R, right. **(B)** The mean values of changed VMHC between patients and healthy HCs.

**Figure 2** displays the spatial distributions of the group mean zVMHC signal values in HCs (**Figure 2A**) and patients with iridocyclitis (**Figure 2B**), respectively. Differences in the VMHC signal values between patients with iridocyclitis and HCs are shown in **Figure 3A**. The mean values of alterations in VMHC between the two groups are shown in histogram format (**Figure 3B**). **Table 3** shows the changed brain regions and corresponding information between patients and HCs. Compared with HCs, patients with iridocyclitis demonstrated significantly reduced VMHC signal values in the bilateral inferior temporal gyrus, calcarine, middle temporal gyrus, and precuneus (voxel- level P<0.01, Gaussian random field correction, cluster-level P<0.05). However, no significant correlations were found between clinical characteristics and the mean VMHC signal values in changed brain regions (all P>0.05).

**Table 3:** Differences in homotopic connectivity between the iridocyclitis group and HC group.

Conditions	Brain regions	MNI			Cluster size	t-score of peak voxel
		x	y	z		
iridocyclitis<HCs	inferior temporal gyrus	-51	-60	-6	120	-4.0742
iridocyclitis<HCs	calcarine	6	-93	24	473	-4.3309
iridocyclitis<HCs	middle temporal gyrus	-66	-24	0	65	-4.1273
iridocyclitis<HCs	precuneus	9	-69	39	40	-3.4674

t-score of peak voxel representing significant differences between the two groups. x, y, z are the coordinates of primary peak locations in the MNI space.

VMHC, voxel-mirrored homotopic connectivity; MNI, Montreal Neurologic Institute; HC, healthy control.

### Machine learning classification results

**Figure 4:**
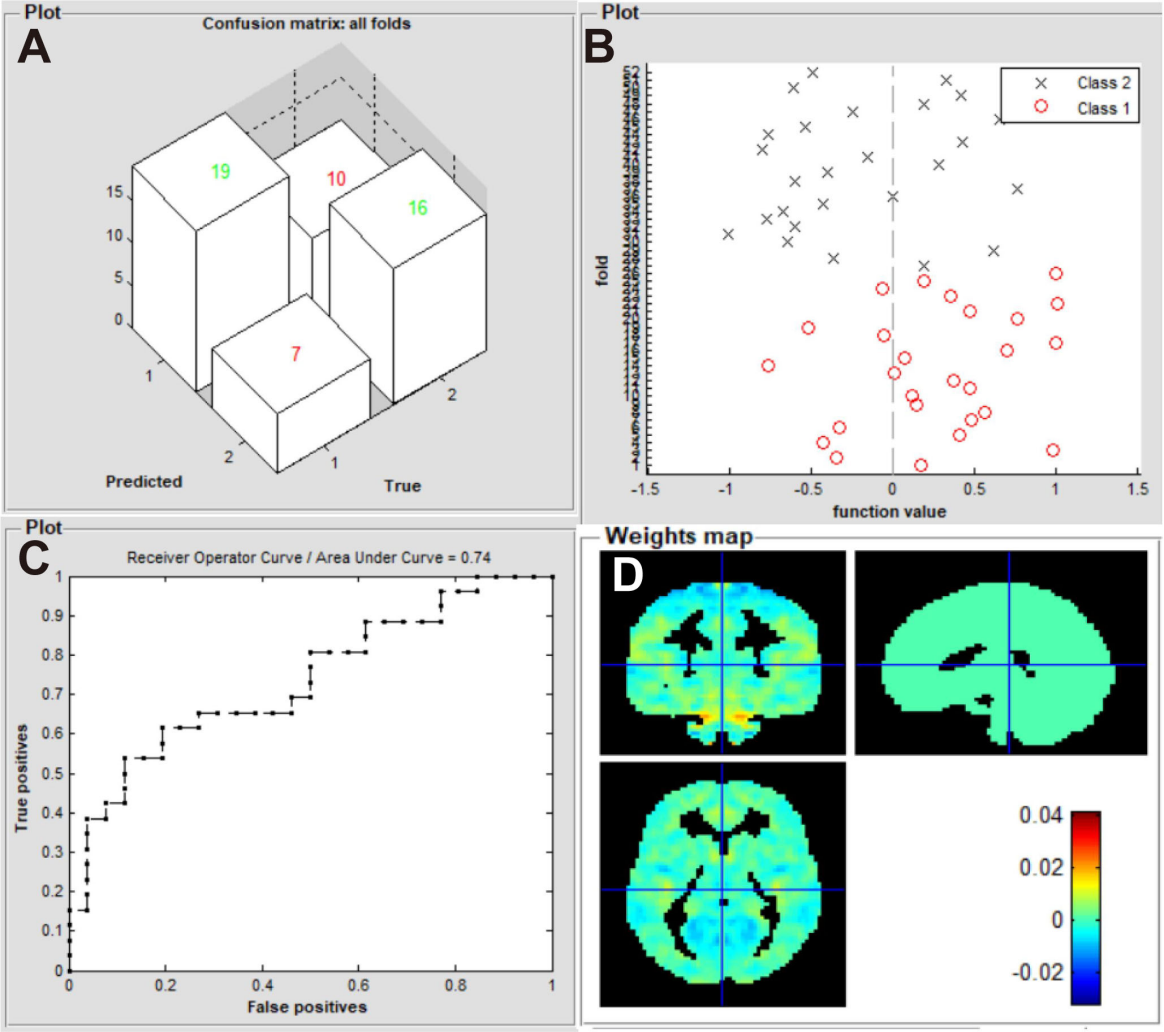
Classification results using machine learning analysis based on zVMHC. **(A)** three-dimensional confusion matrices from SVM classifier; **(B)** function values of two groups (class 1: iridocyclitis; class 2: HC group); **(C)** the ROC curve of the SVM model, and the AUC was 0.74; **(D)** weight maps for the SVM classifier. VMHC, voxel-mirrored homotopic connectivity; SVM, support vector machine; ROC, receiver operating characteristic; AUC, area under the receiver operating characteristic curve; HC, healthy control.

Our machine learning model was evaluated in terms of its ability to identify patients with iridocyclitis and HCs. The SVM classifier applying the LOOCV approach achieved an overall accuracy of 67.31%, sensitivity of 73.01%, and specificity of 61.54% in the binary classification. A receiver operating characteristic curve was generated to assess the model’s performance in terms of discriminating patients with iridocyclitis from HCs; the AUC was 0.74 (P<0.001, non-parametric permutation test) (**Figure 4**).

## Discussion

The VMHC technique is a non-invasive and effective fMRI method for investigating the homotopic connectivity architecture of the brain. This approach has proven successful in neuroimaging, unveiling specific VMHC disruptions in patients with diverse diseases (**Table 4**). This study represents the first exploration of altered homotopic connectivity in patients with iridocyclitis, providing potential insights for the diagnosis and management of this condition. Patients with iridocyclitis exhibited significantly reduced VMHC signal values compared to healthy controls in several brain regions, including the bilateral inferior temporal gyrus, calcarine, middle temporal gyrus, and precuneus (voxel-level P < 0.001, Gaussian random field correction, cluster-level P < 0.05). In addition, our results further support the growing potential of machine learning as a complementary diagnostic tool alongside neuroimaging resting-state data.

**Table 4:** Application of the VMHC technique in ophthalmological diseases.

Author	Year	Disease	Brain regions	
			IC>HC	IC<HC
Huang et al. (17)	2019	late blindness	–	CUN/CAL/LING
Zhou et al. (57)	2018	insomnia	–	MOG/pMTG
Wei et al. (15)	2018	blepharospasm	ITG/IFG/PCC/PosCG	–
Wang et al. (16)	2018	primary open-angle glaucoma	–	BA17/BA18/BA19
Hou et al. (58)	2016	depression	–	SFG/STG/CePL; post- and precentral gyri

IC, iridocyclitis; HC, healthy control, CUN/CAL/LING, cuneus/calcarine/lingual gyrus; MOG/pMTG, middle occipital/posterior middle temporal gyrus; ITG, inferior temporal gyrus; IFG, interior frontal gyrus; PCC, posterior cingulate cortex; PosCG, postcentral gyrus; SFG, superior frontal gyrus; STG, superior temporal gyrus; CePL, posterior cerebellar lobe; BA, Brodmann area.

Our findings indicated reduced VMHC in visual cortical areas. Anatomically, the calcarine sulcus is situated on the medial surface of the occipital lobe, housing the V1. In V1, visual input from each half of the visual field is concurrently relayed to the opposite side. The existence of interhemispheric connections between the visual cortices is an integral feature of visual information integration, potentially playing a role in visual recognition and fusion processes (26, 27). Interhemispheric synchronization of V1 in response to visual stimuli has been investigated through electroencephalography (28–30). In the present study, alterations in VMHC were detected in the bilateral calcarine regions in iridocyclitis patients (31).While iridocyclitis is characterized by inflammation of the ciliary body and iris, morphological studies have unveiled retinal involvement, encompassing retinal thickness, central foveal changes, and alterations in the retinal nerve fiber layer, throughout the progression of the disease (6, 32).; This could lead to reduced light stimulation and decreased retinal input at the visual center, resulting in diverse oscillations of neuronal components. Such variation may impede interhemispheric synchronization, disrupting the processing and transmission of visual information between hemispheres in individuals with iridocyclitis. Consequently, our innovative rs-fMRI evaluation provides additional support for the impact of iridocyclitis on the posterior visual system in patients.

Interhemispheric connectivity was notably decreased in the middle temporal gyrus among iridocyclitis patients compared to healthy controls. This region, along with the inferior parietal, posterior cingulate, anterior cingulate, and medial frontal cortices, is part of in the default-mode network (33)., which remains continuously activated in resting-state conditions (34). The default-mode network is presumably the biological basis of multiple awareness activities, including self-inspection (35), observation of the external environment, and depression (36). Recently, several studies have investigated mental health status and vision-related quality of life in patients with uveitis (37, 38). Maca et al. revealed that patients with human leukocyte antigen-B27 displayed substantial psychopathology in terms of disease coping and depression (39), while Hoeksema et al. discovered that patients with anterior uveitis showed more negative coping strategies and depressive symptoms (40). Therefore, the decrease in VMHC signal intensities between the bilateral middle temporal gyrus in our iridocyclitis patients indicates potential consciousness deficits and dysfunction related to emotional regulation and cognition.

Notably, relevant studies investigated the inflammatory processes and cognitive outcomes linked to the immune-related conditions using MRI techniques. For instance, the atrophy of strategic gray matter regions such as the cortex, hippocampus, thalamus and cerebellum has been identified among the best predictor of cognitive deficits in patients with multiple sclerosis (41). In addition, a growing number of neuroimaging studies have detected abnormal brain activity associated with vision, emotion and cognition of patients with another autoimmune orbital disorder, thyroid-associated ophthalmopathy (42). These findings underscore the potential for similar neurobiological changes in patients with iridocyclitis, suggesting that the immune dysregulation inherent in this condition may also impact brain function. Further exploration of these connections could enhance our understanding of the comprehensive effects of immune-related diseases on both ocular and neurological health.

Additionally, in our study, we noted a reduction VMHC in both the right and left inferior temporal gyrus within the iridocyclitis group. The inferior temporal gyrus resides in the convolution or protuberance of the temporal lobe in each cerebral hemisphere, situated below the middle temporal sulcus and extending towards the inferior sulcus. Previous research identifies this area as the tertiary visual association cortex and a critical hub for language processing. It plays a role in object memory, behavioral learning, and emotional regulation (43). it is also responsible for visual perception, facial recognition, and some cognitive processes (44, 45). Disturbances in this region have been observed in various ophthalmic diseases, including blindness and optic neuritis (46, 47). Therefore, dysfunction in the activities of the bilateral inferior temporal gyrus, in addition to the visual cortex, may result in challenges related to visual memory and item identification.

The precuneus is a superior parietal lobule component that receives visual information from the middle temporal area as the information travels through the dorsal visual pathway. The anterior precuneus is the somatosensory association cortex; it has important roles in object localization, motion detection, and coordination of visuomotor skill (such as arm-eye coordination) (48, 49).The central precuneus is involved in various highly integrated tasks; it is reportedly associated with the default-mode network, episodic memory retrieval, self-processing, and spatial location encoding (50–52). Previous studies have shown that functional abnormalities in this brain region are involved in several vision-impaired ocular diseases, such as anisometropic amblyopia (53), primary open-angle glaucoma (54), and retinal vein occlusion (55). In our study, we found that patients with iridocyclitis exhibited decreased VMHC values in the bilateral precuneus. Thus, we presume that patients with iridocyclitis also experience deficits involving visuospatial imagery and visuomotor coordination.

The use of imaging in diagnosing iridocyclitis remains challenges, as confirmed diagnosis often rely on clinical symptoms. In the last few years, there has been increasing in the use of artificial intelligence for analyzing fMRI data in the field of ophthalmology (56) Combining fMRI with AI holds significant potential for clinical applications by enhancing diagnostic accuracy through the identification of complex brain patterns, enabling predictive modeling for disease progression and treatment responses. In our study, we utilized the SVM approach to analyze zVMHC maps, successfully distinguishing patients with iridocyclitis from healthy controls. We selected a linear kernel for the SVM analysis due to its effectiveness in handling high-dimensional fMRI data. In addition. LOOCV serves as an effective approach to assess model performance and mitigate overfitting based on the relatively small sample size, thereby enhancing the generalizability of our findings. The AUC analysis showed a classification accuracy of 0.74 with the LOOCV method. These results suggest that SVM shows promising classification capabilities with LOOCV. Overall, our findings highlight the sensitivity and validity of machine learning analyses in classifying patients with iridocyclitis by integrating the SVM algorithm with rs-fMRI neuroimaging data.

The present study had several limitations. Firstly, its small sample size may have restricted the statistical power in detecting between-group differences. Future studies with larger sample sizes are essential to validate these effects and provide more robust conclusions. Additionally, we will explore the use of other machine learning models to enhance our analysis. Secondly, the use of a symmetrical template and functional data smoothing in the VMHC technique may not fully account for the brain’s asymmetry. This oversight could mask significant lateralized patterns of connectivity, resulting in an incomplete understanding of the underlying neural mechanisms. Thirdly, the lack of assessment of bilateral gray matter volume or white matter diffusivity could have influenced the functional analysis results. Employing multimodal fMRI techniques, such as resting-state fMRI combined with diffusion tensor imaging, in future studies may enable a more comprehensive analysis of brain structure and function. Lastly, while this study used a cross-sectional design, incorporating longitudinal studies (combined with severity and duration of visual impairment, neuropsychological assessments of patients) would provide deeper insights into the mechanisms underlying interhemispheric alterations and neuropathological changes in patients with iridocyclitis. Future research could focus on tracking changes over time to better understand the progression of these alterations.

## Conclusion

This study is the first to identify of VMHC disorders in patients with iridocyclitis. Our findings suggest that individuals with iridocyclitis display intricate interhemispheric connectivity disruptions within the visual cortex, accompanied by impaired activity in cognition-related regions, such as the default-mode network circuits. These insights highlight the innovative use of VMHC as a biomarker for understanding the neural mechanisms underlying iridocyclitis. In the future, VMHC data could offer valuable insights for the early detection of neuropathological changes associated with this condition, paving the way for more accurate clinical diagnoses and targeted interventions that enhance patient care.

## Data Availability

The raw data supporting the conclusions of this article will be made available by the authors, without undue reservation.
